# Computational Cancer Cell Models to Guide Precision Breast Cancer Medicine

**DOI:** 10.3390/genes11030263

**Published:** 2020-02-28

**Authors:** Lijun Cheng, Abhishek Majumdar, Daniel Stover, Shaofeng Wu, Yaoqin Lu, Lang Li

**Affiliations:** 1 Department of Biomedical Informatics, College of Medicine, The Ohio State University, Columbus, OH 43210, USA; Lijun.cheng@osumc.edu (L.C.); abhishek.majumdar@osumc.edu (A.M.); stover.61@osu.edu (D.S.); wu.2946@buckeyemail.osu.edu (S.W.); 2 Department of Occupational and Environmental Health, School of Public Health, XinJiang Medical University, Urumqi 830011, China; lyq_superior@163.com

**Keywords:** drug recommendation, precision cancer medicine, cancer cells

## Abstract

Background: Large-scale screening of drug sensitivity on cancer cell models can mimic in vivo cellular behavior providing wider scope for biological research on cancer. Since the therapeutic effect of a single drug or drug combination depends on the individual patient’s genome characteristics and cancer cells integration reaction, the identification of an effective agent in an in vitro model by using large number of cancer cell models is a promising approach for the development of targeted treatments. Precision cancer medicine is to select the most appropriate treatment or treatments for an individual patient. However, it still lacks the tools to bridge the gap between conventional in vitro cancer cell models and clinical patient response to inhibitors. Methods: An optimal two-layer decision system model is developed to identify the cancer cells that most closely resemble an individual tumor for optimum therapeutic interventions in precision cancer medicine. Accordingly, an optimal grid parameters selection is designed to seek the highest accordance for treatment selection to the patient’s preference for drug response and in vitro cancer cell drug screening. The optimal two-layer decision system model overcomes the challenge of heterology data comparison between the tumor and the cancer cells, as well as between the continual variation of drug responses in vitro and the discrete ones in clinical practice. We simulated the model accuracy using 681 cancer cells’ mRNA and associated 481 drug screenings and validated our results on 315 breast cancer patients drug selection across seven drugs (docetaxel, doxorubicin, fluorouracil, paclitaxel, tamoxifen, cyclophosphamide, lapitinib). Results: Comparing with the real response of a drug in clinical patients, the novel model obtained an overall average accordance over 90.8% across the seven drugs. At the same time, the optimal cancer cells and the associated optimal therapeutic efficacy of cancer drugs are recommended. The novel optimal two-layer decision system model was used on 1097 patients with breast cancer in guiding precision medicine for a recommendation of their optimal cancer cells (30 cancer cells) and associated efficacy of certain cancer drugs. Our model can detect the most similar cancer cells for each individual patient. Conclusion: A successful clinical translation model (optimal two-layer decision system model) was developed to bridge in-vitro basic science to clinical practice in a therapeutic intervention application for the first time. The novel tool kills two birds with one stone. It can help basic science to seek optimal cancer cell models for an individual tumor, while prioritizing clinical drugs’ recommendations in practice. Tool associated platform website: We extended the breast cancer research to 32 more types of cancers across 45 therapy predictions.

## 1. Introduction

Cancers that appear pathologically similar often respond differently to the same drug. Precision medicine is to deploy effective patient-specific treatments based on the molecular genomic information about the individuals [1,2,3]. Over a dozen therapies have been approved with companion diagnostic tests, assays that predict tumor response to therapy, aimed at assisting in the decision of which therapy strategies should be utilized for specific patients. These include assessments of all kinds of mutation biomarkers for administration of anti-cancer therapies in cancer [4,5,6,7] called the matching molecular alteration strategy for precision medicine. These results have led to increased off-label use of molecularly targeted agents on the basis of identified molecular alterations, such as BRAF V600 mutations for administration of anti-RAF and anti-MEK therapies in melanoma [8], BCR-ABL gene fusions for the use of imatinib in treating chronic myeloid leukemia (CML) and acute lymphoblastic leukemia (ALL) [9], and the loss of PTEN expression for administration of resistance to anti-EGFR monoclonal therapy [10]. However, detection of cancer-related biomarkers is only the first step in the battle. Deciding what therapy options to pursue can also be daunting, especially when tumors can harbor more than one potentially actionable aberration [11]. Further, different mutations/variants in a single gene may have different functional consequences, and response to targeted agents may be context dependent [12]. 

Precision medicine gradually aims toward accurate and effective precise treatment [13] by algorithm guidance. Emerging next generation sequencing (NGS) techniques and therapeutic drugs based on molecular profiling and genomic characteristics will help achieve that goal [12]. The Cancer Genome Atlas (TCGA) project (https://cancergenome.nih.gov/abouttcga/overview) has now provided detailed molecular compositions for over 11,000 cancers from at least 33 anatomic sites which can be used to define cancer subsets, including cell-of-origin patterns, oncogenic processes, and signaling pathways that could identify more homogeneous populations likely to benefit from similar interventions [14]. The Cancer Cell Line Encyclopedia (CCLE) conducts a detailed genome and genetic characterization of a large panel of human cancer cell lines, which collects the whole genome, the whole exome, and the RNA-seq datasets encompassing nearly 1000 human cancer cell lines [15,16]. The pharmaceutical Cancer Therapeutics Response Portal (CTRP) takes a large scale of 664 adherent cancer cell lines from the CCLE to observe 481 small-molecule drugs responses [15]. The results of the largest cancer cell line drug sensitivity data analyses overlapping with clinical practice will lead to the drug most likely to yield the best response for a patient and provide more accurate measures of the drug’s sensitivity for specific patients [3]. Systematic similarity comparisons between cancer cells and patients in multi-omics levels [17] illustrates that gene expression obtained the most signatures with an average correlation of 0.75 whereas DNA mutation level is around 0.15. Gene expression profiles provide the largest amount of information and are apt to disclose cancer cell variation associated with individual tumors. However, the major challenge underlying the emerging precision medicine initiative is how to make links between cancer cell in vitro models and drugs that can be used to guide treatments for individual patients leading to improved outcomes by gene expression profiles for both cancer cells and tumors.

In vitro human cell line models have been widely used for cancer pharmacogenomic studies to predict clinical patient response and to help identify either within or between cancer lineages [3]. Many calculation models for drug sensitivity prediction are set up by connecting these mRNA profiles with drug screening [2,3,4,5], including a community effort to assess and improve drug sensitivity prediction algorithms in breast cancer cell lines [6]. Heiser et al. [7] identifies novel molecular portraits associated to compounds’ responses by using a much smaller panel of 49 breast cancer cell lines with sensitivity to a panel of 77 compounds. However, these models are limited to predicting drug responses in cancer cells only, not for a specific patient’s treatment. A central challenge in drug sensitivity research is to create models that bridge the gap between cancer cells and tissues in which the drug responses are manifested for the single patient. The issue of how to utilize these genome mRNA data with drug responses from cancer cells to guide the optimum drugs’ recommendation at the level of single patient has not been addressed. 

Recently, machine learning technology facilitates precision cancer medicine to detect off-label drug selection on the basis of tumor molecular profiling, but not single biomarker-selected, such as the SHIVA trial [18] achieves precision cancer medicine driven by an algorithm. A bioinformatics approach by pair multi-omics genomics variation is used to detect off-label drug selection for precision medicine among triple negative breast cancer patients successfully [1]. An integrated optimization method is used for identifying the deregulated subnetwork for precision medicine in cancer [19]. PMTDS is based on genetic interaction networks for target-drug selection in precision cancer medicine [20]. These strategies build up their own models by patients’ genome variation. However, there was a lack of available evidence of the detrimental effect of guidance such as in vitro to connect basic science for molecular mechanism detection.

In this paper, an optimal two-layer decision system model is developed to identify the cancer cells that most closely resemble an individual tumor and seek the optimum therapeutic interventions for precision medicine automatically for the first time. Our aim is to glean from increasingly massive cancer cells and associated drug screening, as well as available patient big data sets, insight into the optimal cancer cell models for tumor at the individual level. Those insights could guide the development of new drugs, uncover new uses for old ones, and suggest personalized drug effective prediction. Two important questions are focused on: (a) how to systematically identify optimum cancer cell lines as an individual tumor model by transcriptome genomic comparison; (b) how to identify optimum therapeutic interventions for a single patient based on this drug screening on a large scale. We detected the drug response concordance rate between the real therapeutic drugs in clinic and the drugs selected by our system of identified similar cancer cell lines. Seven drugs, cyclophosphamide, docetaxel, doxorubicin, fluorouracil, paclitaxel, tamoxifen, and lapitinib, were simulated on 315 cancer patients. Another 1097 patients with breast cancer in TCGA were investigated regarding the similar cancer cells and their associated efficacy drugs by the novel computational cancer cell model. Each sub-type of breast cancer, including Luminal A, Luminal B, Her2 positive, and Triple negative breast cancer, were observed in detail. We also extended the breast cancer research to pan-cancer 33 types of cancer across 45 therapy predictions. The associated application platform was developed and can be accessed by the link: https://pcm2019.shinyapps.io/Drug_Response_Prediction/.

## 2. Materials

### 2.1. Cancer Cell Line Transcriptomics and Associated Drug Responses 

We collected molecular mRNA data from the database CCLE, which included 610 gene expression profiles with 20,531 genes [16,18], which were tested by an Affymetrix HU133 PLUS2.0 array (for sample annotation see Tables S1 and S2). The 610 cancer cell lines provided 481 drug sensitivity responses in CTRP [16,21,22] which comprised of 70 FDA approved drugs, 100 experimental compounds, and 311 small molecule probes, about half of which had no identified protein targets. AUC (area under drug response curve) was used to assess the extent of exposure of a drug (all drug annotations are listed in Table S3). Functional genomics data in this manuscript can be found as the following: microarray (Breast tumors and cell lines) from Gene Expression Omnibus (GEO), GSE66305; Gene expression, Copy number variation (CNV), DNA exome mutation sequencing, Reverse Phase Protein Array (RPPA) datasets for breast tumors are from Cancer Genome Atlas (TCGA) Data Portal (https://gdc.nci.nih.gov/); Gene expression of breast cancer cell lines are from Cancer Cell Line Encyclopedia (CCLE) (http://www.broadinstitute.org/ccle); Large scale of drug screening in cancer cells is from the Cancer Therapeutics Response Portal (CTRP, https://portals.broadinstitute.org/ctrp). Datasets supporting the results of this article are included in the additional files.

### 2.2. Patient Transcriptomics and Associated Drug Responses 

The Cancer Genome Atlas (TCGA) is a public database which provides the opportunity to evaluate the predictive utility of molecular data for clinical drug responses in 32 cancer types. We collected 1097 transcriptome expression profiles in breast cancer from TCGA by Genomic Data Commons Data Portal (https://portal.gdc.cancer.gov/), which were sequenced by the platform Illumina HiSeq 2000. We classified these breast cancer patients into five subtypes; Luminal A, Luminal B, Her2, Basal Like, and Others according to the report by Jiang et al. [17] (for patient classification see Table S2).

Molecule-based predictions of drug responses are one major task of precision oncology. The 389 patients with breast cancer had persisted for an average of 2 years following breast cancer treatment of 43 drugs in TCGA and these reports are collected (for detailed information see Table S4). All of these samples are from primary tumor presentations for further mRNA gene expression molecular assessments. One hundred and eight samples out of 389 have clinical annotation information. Based on these records, we removed the patients who received drug treatments prior to tumor resections. The top six therapies used to observe the accordance of drug response sensitivity between the model prediction by cancer cells and the real breast cancer treatment in clinic, include cyclophosphamide (cytoxan, 96 samples), doxorubicin (adriamycin, doxil, 50 samples), paclitaxel (taxol, 42 samples), taxotere (docetaxel, 47 samples), fluorouracil (31 samples), and tamoxifen (18 samples). On the other hand, we collected 31 patients with HER2-positive breast cancer and only treated with Lapitinib from Gene Expression Omnibus (GEO) Datasets Series ID GSE66305. A total of seven drugs across 315 breast cancer patients were used to model accuracy tests of cancer cells in vitro in guiding precision breast cancer medicine in clinic practice. All 315 patients provided the clinical drug response results and associated gene expression profiles before treatment (Table 1, for details see Table S4). Assessment of the change in a tumor after drug treatment is an important feature of the clinical evaluation of cancer therapeutics. We used the responder and non-responder as evaluation of patient sensitivity or insensitivity to each drug, which are useful endpoints by RECIST standard [23] shown in the literature [24,25,26]. Drug response reflects drug sensitivity which means tumor shrinkage. The responder is related to the complete response or the stable disease. Drug non-response reflects non-sensitivity which means disease progression. Non-responder reflects partial response or the progression of a disease. 

## 3. Methods

### 3.1. Data Preprocessing

#### 3.1.1. Gene Annotation and Normalization by Rank

Gene expression profiles of cancer cells and patients performed the following preprocessing before they were used in the subsequent steps. We first filtered out all those genes from the gene expression data that did not have any value. On the other hand, we constructed a histogram by the expression data and removed the genes whose expression located at the lower 5% area of the curve (two sides), which were thought of as outliers of the data. As a result, genes with only non-zero expression values were left. Next, we annotated gene names to standardize symbols for both the patients and the cell-line data by using the reference HUGO (https://www.genenames.org/). These overlapping genes in both datasets were used for data analysis later. These expression data were normalized by rank in descending order for all cancer cell lines and patients. This strategy overcomes any normalization issues while combining data from different sources/platforms. 

#### 3.1.2. Batch Effect Removal and Normalization 

All gene expression profiles were preprocessed by removing batch effect and normalization sequentially by R function *removeBatchEffect* [27]. 

### 3.2. Optimal Two-Layer Decision System Model for Precision Medicine 

**Figure 1:**
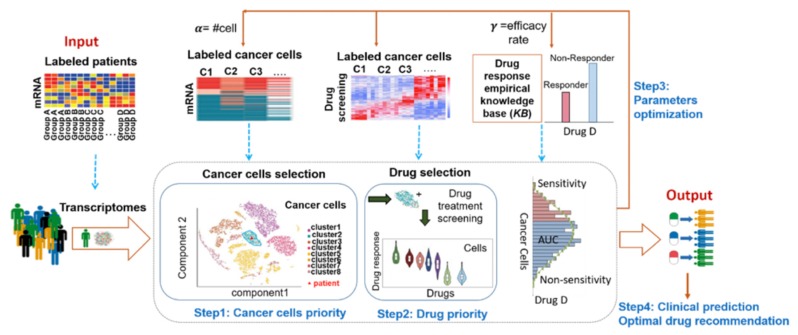
Computational cancer cell model, called the optimal two-layer decision system, to guide precision cancer medicine.

The cancer cell model for precision medicine provides an excellent platform for cancer cell representatives as well as drug selection by comparing it to a cancer cell knowledge base. We hypothesize that drugs that are potent on a cell-line will have a similar effect on a patient if the patient and cell-line transcriptomes are similar to each other. The optimal two-layer decision system model was developed as shown in Figure 1. The model uses a two-layer recommendation system wrapped with an optimal search system to detect optimal cancer cells and efficacy drugs for the right patient by comparing with mRNA profiles and drug response sequentially between cancer cells and an individual tumor. The first layer is to recommend the best-suited cancer cells for a single patient by comparing thousands of single cancer cells’ transcriptome and his/her tumor’s gene expression profile (Figure 1, Step1). The second layer is to recommend the best efficacy drug for the single patient by comparing large-scale drug screenings on these selected cancer cells and patients’ clinical drug empirical knowledge base (Figure 1, Step2). Drug response empirical knowledge base provided prior patients’ records on each drug response from clinical practice and was used for the efficacy rate calculation (*γ*) of specific drug response in a cohort. Efficacy rate (*γ*) becomes the important parameter in connection to the continuous drug response on cancer cells to the binary drug response (Yes or No) on patients. The calculational cell lines model for a patient depends on the setting of the three synchronize parameters, including the number of cells (*α)* and the efficacy rate (*γ*). They will decide the model in accordance with bridging drug screening on cancer cell lines to patients’ drug responses. A grid searching algorithm was designed to seek the optimal parameters on the loop (Figure 1, Step3). When a new patient transcriptome is received as input, the optimal two-layer decision system can predict the most optimal cancer cells and associated efficacy drugs for the patient by the trained model (Figure 1, Step4). 

*Step1: Optimal Selection of Cancer Cells*

This step is essentially a clustering analysis, where our goal is to group cancer cell-lines and the patient (P) tumor into clusters, so that the cluster containing the patient tumor will represent the cancer cells most similar to the patient P. Once such a cluster has been formed, we select an optimal number of cancer cell lines to be recommended for P. Let *α* denote the optimal number of cancer cell lines to be selected. 

We use K-nearest neighbor approach (*KNN*) based on Spearman Correlation measurement to identify the most similar cell-lines to the patient. We calculate the Spearman correlation coefficient value between the patient and each of the cancer cell-lines by entire available genomic data without feature selection using the formula:

(1) $$\frac{\sum \left(\overset{´}{P}-m_{\overset{´}{P}}\right)\left(\overset{´}{CL_{i}}-m_{\overset{´}{CL_{i}}}\right)}{\sqrt{\sum {\left(\overset{´}{P}-m_{\overset{´}{P}}\right)}^{2}\sum {\left(\overset{´}{CL_{i}}-m_{\overset{´}{CL_{i}}}\right)}^{2}}},$$

where $\overset{´}{P}$ is the ranked patient data P, $m_{\overset{´}{P}}$ is the mean of $\overset{´}{P}$, $\overset{´}{CL_{i}}$ is the ranked cell-line data $CL_{i}$ and $m_{\overset{´}{CL_{i}}}$ is the mean of $\overset{´}{CL_{i}}$. We sort the list of cell-lines according to descending order of correlation coefficient values (as higher correlation value indicates higher similarity) and select the top *α* cell-lines. 

*Step2: Optimal Drug Selection of The Selected Cancer Cells*

The area under the curve (AUC) is commonly used to assess the extent of exposure of a drug. A low AUC value indicates higher sensitivity to the drug. 

We will recommend the most similar α cancer cells and the top (the lowest AUC values) efficacy drugs to the patient. Whether drug *D* is sensitive or not to a specific cell is decided by its threshold $\theta_{D}$. 

Whether each drug in cancer cells has a high efficacy (sensitivity) or not is decided by all cells’ AUC distribution and the drug response rate γ of the patient population. Both cancer cells’ and patients’ drug response will decide a threshold $\theta_{D}$ to justify the drug *D*’s efficacy or not to a cancer cell. We use the following steps to calculate the threshold $\theta_{D}$:

1. If *D* is a drug with prior drug response information, then we use our method to obtain the optimal cancer cells for all patients treated with *D*, and combine them into a single unique cell-line list *L*. We also set γ = the fraction of patients that have a response to *D*. If *D* is a drug with no prior information, we set *L* to be the 610 cell-lines from CCLE and γ = 0.5.
2. We create a histogram (*H*) of the different AUC values of *D* across all cell-lines in *L*. We calculate the frequency and cumulative frequency of each interval in the *H*.
3. We calculate percentile point P_γ_ as follows [28]: $P_{\gamma }=\phi +\left(\gamma n-cf\right)/f$, where γ = fraction of patients having a response to *D*, *n* is the total number of cell-lines i.e., |*L*|, *φ* is the lower limit of the interval in *H* containing the γ*n*^th^ AUC value, *cf* is the cumulative frequency up to *φ*, and *f* is the frequency of AUC values in the interval in *H* containing the γ*n*^th^ AUC value.
4. Finally, we compute $\theta_{D}$ as the ceiling value of percentile point P_γ_, $\theta_{D}$ = ⌈P_γ_⌉.

Once the value of $\theta_{D}$ is obtained, we compute the list of drugs to which the patient P will be responsive.

A lower AUC value indicates higher sensitivity to the drug. For each cell line in $CL_{\alpha \_SP}$, we sort the 481 drugs applied to it (from CTRP) in ascending order of AUC values. We remove any entries with missing values. We take the sorted drug list for each cell line in $CL_{\alpha \_SP}$ and combine them into a single unique list of drugs *U*_DP_, called the average of AUC values of each drug across the most similar *α* cell lines ($CL_{\alpha \_SP}$). By ranking the average of AUC values, we prioritize the drugs as the most efficient drugs for the single patient, where each of drug average AUC is less than the threshold $\theta_{D}$.

For instance, let drug *D* appear four times across the sorted drug lists for each of the *α* cell lines in *CL*_αP_, and A_1_, A_2_, A_3_, and A_4_ be those values. Then the average AUC value of *D* in *U*_DP_ is calculated as $\overset{4}{\underset{i=1}{\sum }}A_{i}/freq\left(D\right)$ = $\overset{4}{\underset{i=1}{\sum }}A_{i}/4$ and stored in *U*_AUC_. We sort all of the drugs in the *U*_DP_ based on their average AUC values in *U*_AUC_ in ascending order.

*Step3: Parameter Optimization for High Accordance of Drug Response Sensitivity between The Computational Cancer Cell Model and The Single Patient by Approximating Fitness Function*

Fitness function is used to seek the high accordance of drug sensitivity between the recommended drugs from the calculational cancer cells model cells and the real drug response from patients by adjusting parameters of *α* and *γ*. These parameters connect the drug response sensitivity in cell lines continuous variation AUC to binary variation (responder or not response) in patients directly. A confusion matrix is used for statistical calculation of accordance accuracy (*AC*). Table 2 shows the number of correct and incorrect predictions made by our cellular model compared to the actual patients’ outcomes (response, not response) in the drug response. Performance of the cancer cellular model is commonly evaluated using the data in a 2 × 2 confusion matrix for two classes (Response and Not Response). Here in the accordance evaluation, we defined accordance as ‘Yes’ if a particular drug *D* has the same response status in both the clinical patient record as well as our recommended drug list for patients.

We define an accordance measurement of a drug *D* (*AC_D_*) as the percentage of responses predicted by our model that correctly matches with the recorded response for drug *D* by its prior drug response in cancer population. *AC_D_* will be used to evaluate the accuracy by the computational cancer cell model for a single patient in drug response prediction. For each drug *D* in our knowledge base *KB*, we create a confusion matrix of predicted response and recorded response as shown in Table 3 and calculate its accordance (*AC_D_*) as follows:

*AC_D_* = (a + d)/(c + d).

For instance, let there be 20 patients treated with a drug *D*, out of which 15 are recorded to be responsive and five non-responsive. Now let our method predict correctly 15 patients to be responsive, two patients to be non-responsive, and incorrectly three patients to be responsive. Therefore, the accordance accuracy measure of our method is, *AC_D_* = (15+2)/20 = 85%.

We define our fitness function $F{\left(\alpha \right)}_{D}$ for any drug *D* in our *KB* as follows:

(2) $$F{\left(\alpha \right)}_{D}=\left(\frac{\sum_{j=1}^{\left|P_{D}\right|}\sum_{i=1}^{\alpha }Spearman\_Correlation\_Coefficient\left(cl_{i},p_{j}\right)}{\alpha \left|P_{D}\right|}\right)+AC_{D},$$

where $P_{D}$ is the set of patients treated with drug *D* recorded in *KB*, $p_{j}\in P_{D}$, $cl_{i}\in CL_{p_{j}}$, $CL_{p_{j}}$ is the set of cancer cell lines most similar to patient $p_{j}$, obtained by our method and α∈[1, 61]. The fitness function consists of two parts. The first part in Equation (1) denotes the average Spearman correlation coefficient value of the cell-lines recommended to patients in a typical population $P_{D}$. $CL_{p_{j}}$ is the cell-line sets recommended by our method that are most similar to patient $p_{j}\;\in \;P_{D}$, sorted in descending order of correlation values. The number of cells recommended to each patient is α. The second part in Equation (1) is the accordance measure between the drug *D* response calculated by our computational cancer cell model and the real drug response of patients in the clinic. 

### 3.3. Searching Algorithm to Obtain Optimal Parameter α 

Our fitness function was used to test the accordance of drug response predictions by our cancer cell method and the clinical drug real response to a specific drug. We hoped to seek the highest accordance accuracy between cancer cell models to predict patient drug efficacy with maximal fitness. We designed a simple search algorithm to obtain parameter α under a fixed *γ*, where α is the number of cell-lines that are most similar to patient P; γ denotes the efficacy rate of patients to a specific drug *D* response in a particular cohort, such as triple negative breast cancer. Therefore, the value of *γ* is different for different drugs. Typically, the value of *γ* is obtained from knowledge base *KB*. If a particular drug is not available in *KB*, we assume the value of *γ* for that drug to be 0.5. Our objective was to search for the lowest value of α∈[1, 61], that maximizes the value of *F(*α*)*. We used a 10-fold cross-validation method to calculate the lowest value of α that gives the highest value of $F{\left(\alpha \right)}_{D}$ as well as the highest cross-validation accuracy value for all seven validation drugs. 

### 3.4. Default Value of Parameters α and γ

Given a specific drug *D*, if the drug *D* is not in *KB* (no prior information), the value of γ is considered to be 0.5 default. That is, we assume about 50% of patients will respond to the drug *D*. 

We calculated the threshold values for the seven drugs in *KB* using the default value of α = 5% of total cell-lines (610*0.05) to be 30.

## 4. Results

### 4.1. Optimal Cancer Cells and Drug Selection for Precision Cancer Medicine 

We simulated our computational cancer cell model on clinical trial data results for seven FDA approved drugs, cyclophosphamide, docetaxel, doxorubicin, fluorouracil, paclitaxel, tamoxifen, lapitinib; six from TCGA, and one from GEO. CCLE and CTRP cancer cells were integrated with TCGA and GEO data for model construction and drug recommendation (Table 1). Therefore, when a new (incoming) patient comes in, our model can predict the response result for the 481 drugs mentioned in CTRP for that patient. Our model provides an ordered list of drugs from higher to lower sensitivity. We further divide the resultant drug list into four distinct categories: (1) FDA Approved: drugs which are approved by FDA; (2) Clinical: drugs which are already in use (Phase 1/2/3/4) in some kind of clinical trial/trials; (3) PreClinical: drugs which are involved in some of sort of non-human trials or animal models; and (4) Others: drugs which do not fall into the first three categories. This way, the user has a clear impression of the different categories of drugs that will be most responsive for patient P. We include our complete list of FDA approved drugs and clinical trial drugs in Tables S14 and S15.

Our model makes use of our drug response knowledge base (*KB*) on TCGA patients shown in Table S5, where we consider the seven drugs. Our knowledge base basically records the percentage of patients that are responders for each of these seven drugs. Table 1 describes the response rate γ as prior knowledge. The number of cell-lines α, to be used by the model for predicting the drug response rate is determined by parameter optimization.

#### 4.1.1. Optimal Parameters Selection and Accordance Validation Between Cancer Cells and Tumor to Drug Sensitivity

We simulated our calculational cancer cell model on seven drugs to search the optimal value of the parameter α using 10-fold cross-validation. Our objective was to keep the value of *α* as low as possible while getting the maximum fitness and maximum cross-validation accuracy across all seven validation drugs, where *α* represents the size of cancer cell-line list recommended to be most similar to the patient P and *γ* represents the response rate of individual drug. The parameter *γ* was derived from existing knowledge. In case of the seven validation drugs, *γ* was derived from the knowledge base (*KB*). In cases of drugs with no prior information i.e., outside the *KB*, a default value of 0.5 was selected. This can be changed by the user. Therefore, only parameter *α* can be subjected to optimization. We used 10-fold cross-validation to search for the lowest value α∈[1, 61] that maximizes $F{\left(\alpha \right)}_{D}$ across all seven validation drugs. We found that our cross-validation gave the optimal value of *α* to be 30.

#### 4.1.2. Validation of Drug–Patient Response Accordance in Clinical Practice

We used Fisher’s exact test F1 to ascertain the association of the response for each drug in our knowledge base. The contingency table for each of the seven drugs was set up to ascertain the statistical significance of the association of the performance of the drugs suggested by our method and those in the TCGA/GEO response dataset is shown in Table 3. It provides the Fisher’s exact test statistic $\chi^{2}$ value and $AC_{D}$ for all seven drugs. For a two-tailed test we considered the tables with extreme, in-accordance studying proportions, that the probability is *p*-value. The exact Chi-squared $\chi^{2}$ *p*-value for all drugs is greater than 0.05. Fisher’s exact test results show that our model’s prediction of drug performance is not random and is in accordance with the recorded data. 

We validated the results of our prediction method using clinical trial data results of these FDA approved seven drugs. We found that our prediction method has a high average accordance (> 90.8%) across all seven drugs. In the case of the drug fluorouracil, the accordance is 100%. For the drugs cyclophosphamide, docetaxel, doxorubicin, and paclitaxel, our accordance value is over 91%. 

## 5. Application

### 5.1. Optimal Cancer Cells and Drug Selection for Precision Breast Cancer Medicine

We applied our method to 1097 patients with breast cancer to identify the most similar cancer cells. These cell lines can then serve as tumor models to select optimal drugs. Detailed description of each patient, their cell lines, and their recommended drugs can be found in Table S6. We classified the different TCGA patients according to their cancer subtypes as provided in the paper by Jiang et al. [17]. 

### 5.2. Drug Recommendation Level Separation

The goal of precision medicine is to enable clinicians to quickly, efficiently, and accurately predict the most appropriate course of action for a patient. To overestimate the potential benefit of molecularly guided cancer drugs, we do have the sequential recommendation, first FDA approved oncology drugs, followed by the clinical trial delivery, pre-clinical drugs, and then other type of perturbations. Online Drug Repurposing Hub (http://www.broadinstitute.org/repurposing) contains a comprehensive library of clinical compounds suitable for testing. Drug Repurposing Hub [29] is a hand-curated collection of 6113 compounds, experimentally confirming their identities, and annotating them with literature-reported targets. The detailed data can be found in Tables S14 and S15. In which, it includes 2362 that are FDA approved, 1618 drugs that are marketed around the world or that have been tested in human clinical trials, 2036 preclinical drugs, and 96 withdrawn (others). On the other hand, we collected 141 FDA approved drugs for different types of cancer from the National Cancer Institute (NCI, (https://www.cancer.gov/about-cancer/treatment/drugs). All drug names were standardized by DrugBank ID (https://www.drugbank.ca/) annotation.

### 5.3. Identification of The Similar Cancer Cells for Individual Patients with Breast Cancer 

For each cancer subtype, we sought similar cell-lines (Spearman correlation method) and their recommended drugs by our computational cancer cell model. The top three most frequently used cancer cell lines for that group of patients is displayed in Table 4. Treatment recommendations were generated using our algorithms. We list the top eight most frequently used drugs in all four categories, FDA Approved, Clinical trial, Pre-Clinical, and others for that group of patients in Table 5. By design, the tool is compatible with the workflow of clinicians and also simplifies the process of drug selection and managing the biological complexity that underlies cancers for them. 

### 5.4. Identification of The Similar Cancer Cells and Associated Optimal Drugs for Patients with Triple Negative Breast Cancer 

**Figure 2:**
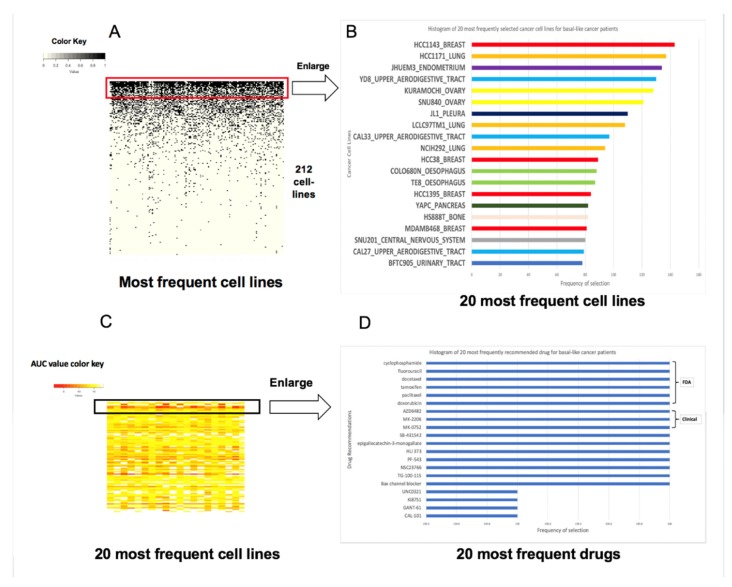
The most frequently occurring cancer cells and recommended drugs to 156 basal like patients with breast cancer. (**A**) The distribution of the different cell lines selected by our method for patients with basal like breast cancer. The *X*-axis contains the different patients while the *Y*-axis consists of the different cancer cell lines. The cell lines were sorted based on the frequency of their selection in descending order. (**B**) An enlargement of the top 20 cancer cell lines with the highest frequency. (**C**) A heatmap distribution of the drugs for basal like cancer patients based on area under the curve (AUC) values. The *X*-axis is the cancer cell lines that are selected from these patients and the *Y*-axis is the different recommended drugs. (**D**) A frequency depiction of the top 20 recommended drugs to these patients.

Triple negative breast cancer (TNBC, Basal like) comprises 15–20% of all breast cancers. It is defined by the lack of estrogen receptor (ER) and progesterone receptor (PR) expression, and a normal human epidermal growth factor receptor 2 (HER2) gene copy number and expression but few treatment options exist outside of chemotherapy [1]. While TNBC is widely held to be particularly aggressive and lethal, the majority of patients with early-stage TNBC will never experience a distant metastatic recurrence or die from their disease. Based on these reasons, we focus on TNBC drug repurpose study and identify the similar cancer cells and associated optimal drugs for TNBC patients. At the same time, we compare the treatment results in more detail in clinical practice. The most frequently occurring cancer cells and recommended drugs to 156 basal like patients with breast cancer is observed in Figure 2. 

Two hundred and twelve cancer cells display correlation to TNBCs (Figure 2A). The three most frequently similar cancer cells to TNBC patients are HCC1143 (breast cancer cell), HCC1171 (Lung cancer cells), and JHUEM3 (endometrium cancer cell), however they do not all originate from breast cancer cells although some factors behind the result are unknown (Figure 2B). We compared the current result from the Spearman correlation measurement where the average correlation coefficient values were 0.83, 0.85, and 0.87 to 156 TNBCs under background correlation value of 0.53 of all cancer cells.

Low AUC values indicate high sensitivity to the drugs based on which drug selection is performed. The drugs were sorted based on frequency of recommendation in descending order to these fit cancer cells. As expected, the lowest AUC values appear at the top corresponding to the top most frequently recommended drugs. The AUC values increase down the *Y*-axis coinciding with the drugs that are selected at a lower frequency (Figure 2C,D). The topmost frequently recommended drugs to TNBC include six of the drugs from our knowledge base (*KB*) as well as other drugs such as AZ6482, MK2206, SB-431542, GANT-61, and so on.

Fischer’s exact test shows that our expected response of a drug is not random, but in accordance with the performance as found in the TCGA response dataset. Clinical validation and implementation further validate our methods’ accuracy and reliability, although there are similarities within other types of cancer cells as well as within breast cancer (Table 3, Table S5). Tables S7–S13 show the validation results of drug response prediction accordance between our method and documented clinical treatment response in the same patients in detail. There might be two possible explanations for this: (1) these non-breast cell-lines might be truly highly correlated with breast cells in which case it might be fruitful to investigate the underlying reason for it, and (2) there might be some contaminations in the patient tumor sample collected which pulls out these non-breast cell-lines in our results. We also see the same six drugs from our knowledge base (*KB*) consistently appearing as the recommended option for FDA approved drugs across all five subtypes. This might be attributed to the fact that all three drugs are chemotherapy-based drugs that have high sensitivity in patients (have low AUC values), that makes them an easy choice for breast cancer patients only if drug efficacy is considered. We also present lists of other categories of drugs for the different subtypes of breast cancer patients.

## 6. Conclusions

The novel drug response prediction model for optimal cancer cell selection is used to recommend efficacy drugs for individual patients. Accordingly, an optimal grid parameter selection was designed to seek the highest possible accuracy in guiding drug selection efficacy from in vitro cancer cells to the patient. Based on the model, we can observe the similarity of cancer cells to the patient directly, at the same time, simulate optimized drug recommendations to the single patient. The optimal two-layer decision system overcomes the difference challenge in comparison to the heterology tumor and cancer cells, and continual variation of drug response in vitro in comparison with discrete ones in clinical practice. Our novelty lies in using the complete genomic data of a single patient to match with cancer cells in conjunction to providing drug recommendations to the patient. Our method is robust in that it can accommodate any type of mRNA profiles when searching for similarities between cancer cells and the patient by rank of gene comparisons, a non-parameter measurement, but not the mRNA value to overcome the profiles’ platform differences. 

We simulated our algorithm on clinical trial data for seven drugs from TCGA and GEO datasets. Validation results reveal that our prediction method has an overall accordance accuracy of >90.8% for seven drugs, cyclophosphamide, docetaxel, doxorubicin, fluorouracil, paclitaxel, tamoxifen, and lapitinib. These cancer patients whose therapeutic drugs are concordant with drugs selected from our approach of cancer cell have better survival outcome than those who do not with 90.8% in accordance. We detected the most similar cancer cells and optimal drugs for four subtypes of breast cancer Luminal A, Luminal B, Her2, and Basal Like (triple negative breast cancer, TNBC) in the 1097 patients with breast cancer in TCGA, especially for TNBC in detail, which can be more challenging to treat, without any small molecular inhibitors in clinic currently. 

It is the first time a computational cancer cell model to guide efficacy drug treatment for individual cancer patients has been developed. The model constructs an important bridge to connect basic science and clinical translation study. This unique feature of the system model will help users, including doctors and biologists, to recommend both the optimum cells and drugs suited for individual patients by flexible molecular feature input and gene expression profile. If a new patient P is to be treated, our method can predict whether the patient will be responsive to a particular drug or not. Our method provides a list of drugs divided into four distinct categories, FDA approved drugs, clinical trials drugs, preclinical drugs, and others, that are presented in descending order of sensitivity to the patient P in an individual category. With this information in hand, the user can make an informed decision about the treatment of the patient. In this current version, our model is only limited to utilizing genomic expression of patients as input to predict drug sensitivity. However, we are working on an updated version that includes mutation single-nucleotide polymorphisms (SNPs) and copy number variation (CNV) data as well as input. Our next paper will present an updated version of the model that will take gene expression (GE) data, mutation SNPs data, and CNV data separately or in any combination as input and then predict the sensitivity of drugs to the patient. The model will also incorporate other forms of cancer treatment.

## Figures and Tables

**Table 1:** Transcriptome before drug treatment and patient drug response after drug treatment, collected from The Cancer Genome Atlas (TCGA) and Gene Expression Omnibus (GEO).

Sources	Drugs	#Patients (Responder, Non-Responder)	Responder Rate	Transcriptome	#Genes
**TCGA**	Cyclophosphamide	96 (93,3)	96.875	HiSeq 2000 Illumina RNA-seq	16,782 genes
	Docetaxel	47 (43,4)	91.489		
	Doxorubicin	50 (47,3)	94		
	Fluorouracil	31 (31,0)	100		
	Paclitaxel	42 (39,3)	92.857		
	Tamoxifen	18 (15,3)	83.33		
**GEO** **GSE66305**	Lapitinib	31 (8,23)	25.8	Affy-HU133 Plus 2.0 Array	54,675 probe sets
**Total**		**315**			**16,780**

Note: Non-responder denoted partial response or clinical progressive response to drug; responder (sensitive) denoted stable disease or complete response to drug.

**Table 2:** Contingency table for drug response comparison between cellular model prediction and true drug response in patients.

Confusion Matrix		Clinical Patient Record			
		Response	Non-Response		
Our Model Prediction	Response	a	b	Positive Predictive Value	a/(a+b)
	Not-Response	c	d	Negative Predictive Value	d/(c+d)
		Sensitivity	Specificity	Accuracy = (a + d)/(a + b + c + d)	
		a/(a+c)	d/(b+d)		

**Table 3:** Contingency table to compare drug response accordance between the prediction and real values for seven drugs.

Drugs	Types	Response	No Response	$\chi^{2}\mathrm{value}$ (*p*-value)	Accordance (%)
Cyclophosphamide	Actual Predicted	93 96	3 0	0.2461	96.875
Docetaxel	Actual Predicted	43 47	4 0	0.117	91.489
Doxorubicin	Actual Predicted	47 50	3 0	0.2424	94
Fluorouracil	Actual Predicted	31 31	0 0	1	100
Paclitaxel	Actual Predicted	39 42	3 0	0.241	92.857
Tamoxifen	Actual Predicted	15 18	3 0	0.2286	83.333
Lapitinib	Actual Predicted	8 3	23 28	0.1822	77.419
**Average**					90.85

**Table 4:** The most frequent three cancer cell lines for 1097 patients in five subtype breast cancer patients.

Types	Luminal A (605)	Luminal B (106)	Her2 (40)	Basal Like (156)	Others (190)
**Cell-lines**	HCC1171_LUNG (569/605)	MDAMB361_BREAST (102/106)	MDAMB361_BREAST (39/40)	HCC1143_BREAST (143/156)	KURAMOCHI_OVARY (171/190)
	MDAMB361_BREAST (558/605)	ZR7530_BREAST (101/106)	ZR7530_BREAST (37/40)	HCC1171_LUNG (137/156)	MDAMB361_BREAST (166/190)
	ZR7530_BREAST (558/605)	YD8_UPPER_AERODIGESTIVE_TRACT (99/106)	YD8_UPPER_AERODIGESTIVE_TRACT (36/40)	JHUEM3_ENDOMETRIUM (134/156)	YD8_UPPER_AERODIGESTIVE_TRACT (166/190)

**Table 5:** The most frequent recommended drugs for 1097 patients in five subtype breast cancer patients.

Types	FDA Approved	Clinical	Pre-Clinical	Others
**Luminal A**	cyclophosphamide	AZD1480	UNC0321	Bax channel blocker
	doxorubicin	MK-0752	UNC0638	TG-100-115
	paclitaxel	AZD6482	GANT-61	PF-543
	tamoxifen	PX-12	RITA	Ch-55
	docetaxel	MK-2206	SB-431542	ZSTK474
	tretinoin	canertinib	AM-580	AC55649
	fluorouracil	tacedinaline	A-804598	NSC23766
	nilotinib	Serdemetan	PIK-93	RG-108
**Luminal B**	cyclophosphamide	AZD6482	GANT-61	Bax channel blocker
	doxorubicin	MK-2206	A-804598	AC55649
	nilotinib	MK-0752	UNC0321	TG-100-115
	paclitaxel	tacedinaline	RITA	NSC23766
	tamoxifen	canertinib	UNC0638	PF-543
	docetaxel	serdemetan	AM-580	BRD-M00053801
	tretinoin	PX-12	SB-431542	C6-ceramide
	fluorouracil	AZD1480	PIK-93	Ch-55
**Her2**	cyclophosphamide	AZD6482	GANT-61	BRD-K29313308
	doxorubicin	canertinib	PIK-93	CAL-101
	afatinib	PX-12	UNC0321	Bax channel blocker
	nilotinib	MK-2206	RITA	MI-1
	paclitaxel	GDC-0941	necrostatin-1	BCL-LZH-4
	erlotinib	AZD1480	UNC0638	AC55649
	gefitinib	MK-0752	SB-431542	TG-100-115
	tamoxifen	saracatinib	FGIN-1-27	NSC23766
**Basal like**	cyclophosphamide	AZD6482	SB-431542	Bax channel blocker
	doxorubicin	MK-2206	GANT-61	TG-100-115
	paclitaxel	MK-0752	UNC0321	NSC23766
	tamoxifen	birinapant	UNC0638	PF-543
	docetaxel	PX-12	PIK-93	HLI 373
	fluorouracil	canertinib	RITA	epigallocatechin-3-monogallate
	nintedanib	saracatinib	necrostatin-1	CAL-101
	vorapaxar	OSI-027	TGX-221	Ki8751
**Others**	cyclophosphamide	AZD6482	SB-431542	Bax channel blocker
	doxorubicin	canertinib	UNC0321	TG-100-115
	paclitaxel	PX-12	RITA	PF-543
	tamoxifen	MK-2206	UNC0638	RG-108
	ibrutinib	MK-0752	AM-580	Ch-55
	docetaxel	AZD1480	necrostatin-1	WZ4002
	tretinoin	GDC-0941	GANT-61	PRIMA-1-Met
	fluorouracil	tacedinaline	PIK-93	ZSTK474

## Supplementary Materials

The following are available online at https://www.mdpi.com/2073-4425/11/3/263/s1, Table S1: The list of TCGA breast cancer samples with associated subtype and clinical data annotations, Table S2: CCLE cell lines annotation, Table S3: Drug annotation in CTRP, Table S4: Drug response to breast cancer patient in TCGA, Table S5: Details about the seven drugs used for validation, Table S6: Our method election results for optimal cells and drug recommendations to 1097 patients with breast cancer in TCGA, Table S7: Validation results for Cyclophosphamide, Table S8: Validation results for Docetaxel, Table S9: Validation results for Doxorubicin, Table S10: Validation results for Paclitaxel, Table S11: Validation results for Fluorouracil, Table S12: Validation results for Tamoxifen, Table S13: Validation results for Lapitinib, Table S14: FDA approved drugs from NCI (version 201708 collection), Table S15: Drug knowledge base from Drug Repurposing Hub (version 20180516).
